# Towards Sustainable Orthodontics: Environmental Implications and Strategies for Clear Aligner Therapy

**DOI:** 10.3390/ma17174171

**Published:** 2024-08-23

**Authors:** Monica Macrì, Vincenzo D’Albis, Raffaele Marciani, Matteo Nardella, Felice Festa

**Affiliations:** Department of Innovative Technologies in Medicine & Dentistry, University “G. D’Annunzio” of Chieti-Pescara, 66100 Chieti, Italy; vincenzo.dalbis@studenti.unich.it (V.D.); ffesta@unich.it (F.F.)

**Keywords:** clear aligner, polymers, plastic pollution, sustainability, thermoforming, orthodontics

## Abstract

The increasing concern over environmental sustainability has prompted various industries to reassess their practices and explore greener alternatives. Dentistry, as a significant contributor to waste generation, is actively seeking methods to minimize its environmental footprint. This paper examines the environmental implications of clear aligner therapy (CAT) in orthodontics and explores strategies to prioritize sustainability in aligner manufacturing and usage. CAT has gained popularity as a viable alternative to traditional fixed appliances due to advancements in biomaterials and computer-aided design (CAD) and manufacturing (CAM) technologies. The global market for clear aligners is expanding rapidly, with significant growth projected in the coming years. To address these challenges, this paper proposes adopting the principles of reduce, reuse, recycle, and rethink (4Rs) in orthodontic practices. Strategies such as minimizing resource consumption, incorporating recycled materials, and promoting proper aligner disposal and recycling can significantly reduce environmental harm. This paper explores emerging technologies and materials to mitigate the environmental impacts of CAT. Additionally, initiatives promoting aligner recycling and repurposing offer promising avenues for reducing plastic waste and fostering a circular economy. In conclusion, while CAT offers numerous benefits in orthodontic treatment, its environmental impact cannot be overlooked. By implementing sustainable practices and embracing innovative solutions, the orthodontic community can contribute to a more environmentally conscious future while continuing to provide quality care to patients.

## 1. Introduction

According to the Eco-Dentistry Association (EDA), dental practices generate substantial waste each year, including 4.8 million lead foils, 28 million liters of hazardous X-ray fixer, 3.7 tons of mercury, 1.7 billion sterilization pouches, and more than 680 million chair barriers, light handle covers, and patient drapes. Furthermore, the average dental office uses approximately 57,000 gallons of water annually. Reducing this environmental impact is a critical issue that spans all industries, leading the dental sector to explore methods to lessen its ecological footprint [1].

Advancements in biomaterials, computer-aided design (CAD), and manufacturing (CAM) have made clear aligner therapy (CAT) a viable alternative to traditional fixed appliances in orthodontics. Over the past decade, demand for CAT has surged, with the global clear aligners market expected to grow from USD 3.1 billion in 2021 to USD 11.6 billion by 2027, representing a compound annual growth rate (CAGR) of 13% [2].

Today, we identify two primary methods: thermoforming on pre-printed models and direct 3D printing [3].

This article aims to explore the environmental implications of aligners while highlighting sustainable operational approaches. We will emphasize strategies to reduce resource usage and waste, examine potential recycling processes, and consider the creation of circular economies, all with the goal of promoting ethical and environmentally responsible practices [4].

## 2. Materials and Methods

The bibliographic research in this study was conducted using PubMed and Google Scholar. The keywords “clear aligner”, “polymers”, “plastic pollution”, “sustainability”, “orthodontics”, and “thermoformation” were employed in both individual and combined searches, utilizing Boolean operators such as AND and OR. A total of 582 articles were identified during the search process. Of these, 45 articles were initially chosen based on a preliminary assessment of their titles. Only articles published in the last 10 years were considered. After reviewing the abstracts, 31 articles were selected for a more in-depth examination. Finally, a refined selection of 27 articles was carried out to compose the research article. In addition, searches were conducted online on specialized websites related to the topic.

## 3. Modern Environmental Issues

The Organisation for Economic Co-operation and Development (OECD) serves as a platform and knowledge center for data, analysis, and public policy best practices. Collaborating with over 100 countries globally, the OECD aims to promote stronger, fairer, and cleaner economies. Their research indicates that annual plastic production has escalated from 234 million tons in 2000 to 450 million tons in 2019. Europe is the second-largest producer, with an average of approximately 114 kg per person. Currently, only 9% of all plastic is recycled: 19% is incinerated, 50% is placed in sanitary landfills, and the remaining 22% is improperly managed, ending up in uncontrolled dumpsites, open pit fires, or leaking into the environment [5].

The World Economic Forum estimates that every minute, a truckload of plastic waste is dumped into the ocean. This plastic debris is carried by ocean currents, often traveling vast distances [6]. The deterioration of plastics generates microplastics; micro- and nanoplastics have been found in foods and beverages served in plastic containers [1].

It has been said that if we do not stop polluting the world’s oceans, there will be more floating bottles than fish in the oceans.

The use of plastic materials is essential to fulfill our daily needs, and the demand for plastics continues to grow. To address this critical issue, the European Parliament has implemented a ban on single-use plastics, such as cotton buds, straws, and stirrers, effective for all member states starting in 2021. Furthermore, member states are required to achieve a 90% collection rate for plastic bottles by 2029, and plastic bottles must include a minimum of 25% recycled content by 2025 and 30% by 2030 [7].

Multiple research papers have reported the existence of microplastic particles in the internal organs and bodily fluids of mammals. The potential harmful effects of ingesting and inhaling microplastics have not been extensively studied, mainly due to the difficulties in accurately measuring and identifying the levels at which nano- and microplastics (NMPs) accumulate in human tissues. The techniques used in these studies are limited by resolution and largely incapable of identifying particles < 1 μm; in addition, they cannot be used to accurately estimate microplastic mass concentration.

A study conducted in USA, the first of its kind, analyzed 62 human placentas, and significant amounts of microplastics were found in all the tissues analyzed. Marcus A. Garcia et al. [8] found that NMPs can accumulate in placentae at levels far higher than was measured in blood.

The materials found with a significant quantity are polyvinyl chloride (PVC), styrene butadiene (SBR), nylon 6 (N6), polyethylene (PE), polycarbonate (PC), acrylonitrile butadiene styrene (ABS), polypropylene (PP), polyurethane (PU), polyethylene terephthalate (PET), polystyrene (PS), nylon 66 (N66), and poly methyl methacrylate (PMMA).

Unfortunately, many of these are also present in aligners [8].

## 4. Orthodontic Pollution

Dentistry is also called into question. Aligners are new tools in orthodontic practice. Clear plastic aligners mainly contain petroleum-based polymers that release various nanoplastics.

The environmental impact of aligner plastics is becoming a significant concern that can no longer be ignored.

In recent years, the demand for personalized dental aligners has significantly increased, particularly among teenagers. Invisalign clear aligners, developed by Align Technology, exemplify this trend and have achieved worldwide popularity. Approved by the Food and Drug Administration (FDA), these aligners had been used by 10.9 million people globally by 2020, with approximately 413,700 cases shipped that year. More than five million teenagers have opted for Invisalign for their orthodontic treatment [9].

To date, over 12.8 million people have successfully improved their smiles using thermoplastic aligners [10].

The weight of each pair of aligners with their bag is about 4.3 g; on average, a treatment is carried out with around 30–40 aligners, and considering only the treatments performed up to 2020, approximately 1875 tons of these materials were produced, of which the majority is medical waste with an infectious risk.

Transparent plastic aligners are mainly composed of PET (polyethylene terephthalate), PETG (polyethylene-terephthalate-glycol), or TPU (thermoplastic polyurethane), as well as other petroleum-based polymers that release a wide variety of nanoplastics.

These nanoplastics that enter our intestines possess the potential to penetrate cell membranes and cause cell destruction or mutation.

The risk of cross-contamination of infections should also be considered, as aligners are thrown into the common garbage without special care after intraoral use [11].

Therefore, the profile of dentistry for the next decade should be shaped by technological innovations, market expectations, and the policy guidelines of the European/world context [12].

The research task of understanding the amount of waste generated from failed prints and post-processing is essential, as well as understanding the kind and amount of the micro- and nanoplastics that may be produced from the fabrication and application of the aligners.

There is a significant gap in our understanding of the improper disposal of plastic aligners, also caused by the increasing trend of using homemade aligners and the resulting consequences for the environment after patient treatment is completed [13].

While saliva and natural oral processes may slowly degrade plastic, ignoring the potential for harmful leaching when these aligners are discarded would be imprudent [14].

## 5. Clear Aligner

### 5.1. Brief History

In the last decades, the demand for treatment with transparent aligners has increased as an alternative to traditional fixed orthodontics; this is presumably due to increased aesthetic needs, more adults approaching orthodontic therapy, and a capillary and efficient marketing campaign [15].

The origin of the aligners can be traced back to HD. Kesiling in 1945; his pioneering intuition was to use a series of thermoplastic tooth positioners to move the misaligned teeth progressively. Later, other contributions were made by Nahoum Ponitz, McNamara, Sheridan, and Truax [3].

However, it was only in 1998, with the introduction of the Invisalign aligner system by AlignTechnology, Santa Clara, CA, (USA), that the advent of modern aligners took place. Invisalign was the first to use transparent thermoplastic polymers and integrated modern CAD/CAM technologies.

In the last two decades, the aligner market has expanded and evolved, both from a commercial point of view, with the birth of numerous new brands (almost twenty in Italy alone), and from a constitutive point of view, with new indications and characteristics [16].

Fundamental elements that characterize the performance, and not only of the aligners, are the composition of the material used for the production and the actual process used for the manufacture; currently, we distinguish two main methods, thermoforming on pre-printed models and the direct 3D printing of aligners [3,11].

### 5.2. Aligner Fabrication Process

The aligner company develops a virtual representation, simulating the series of movements required to achieve the treatment objectives established by the orthodontist. This tool enables the orthodontist to incorporate any necessary adjustments to craft a sequence of dental movements that is both realistic and predictable while also taking into account anatomical limits. This simulation illustrates the quantity of aligners deemed necessary to achieve comprehensive correction of the malocclusion.

Three-dimensional printing technologies are advanced manufacturing methods that use CAD digital models to automatically create personalized three-dimensional objects. These printing technologies can be classified into three main categories: Powder Bed Fusion (PBF), light curing, and Fused Deposition Modeling (FDM). These categories are further subdivided into specific technologies, each with their own distinct advantages. For dental aligners, the second method, light curing, is used [17,18].

**Figure 1:**
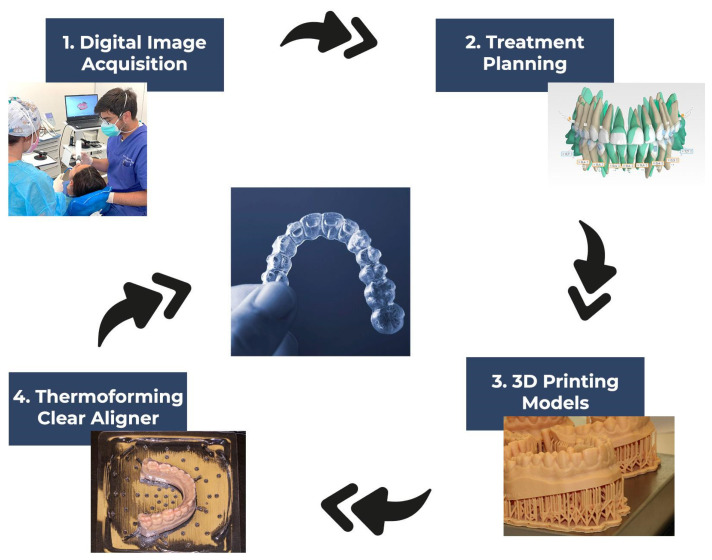
Workflow of fabrication of clear aligners by the homemade thermoforming process.

The thermoforming manufacturing method is the most commercially and clinically adopted manufacturing process for the production of aligners. It consists of printing physical models, as many as the masks that involve the treatment, by which the aligners are obtained with the method of vacuum thermoforming (Figure 1).

The polymers are either individual or blended, and most of the ones utilized for commercial clear plastic orthodontic aligners are polyester, polyurethane (PU) or co-polyester, polypropylene (PP), polycarbonate (PC), ethylene vinyl acetate, and polyvinyl chloride (PVC).

However, the current workflow for manufacturing a series of aligners obtained by thermoforming is voluminous, lengthy, expensive, and contributes to the physical problem of plastic dumping; however, there are no studies or even data on the real quantity of these products [11,19].

Generally, a case with aligners consists of several masks, each worn for approximately 1–2 weeks. At the end of the treatment, the masks do not face any recycling process but are often eliminated in the garbage, increasing the dispersion of these plastics into the environment [11,12].

**Figure 2:**
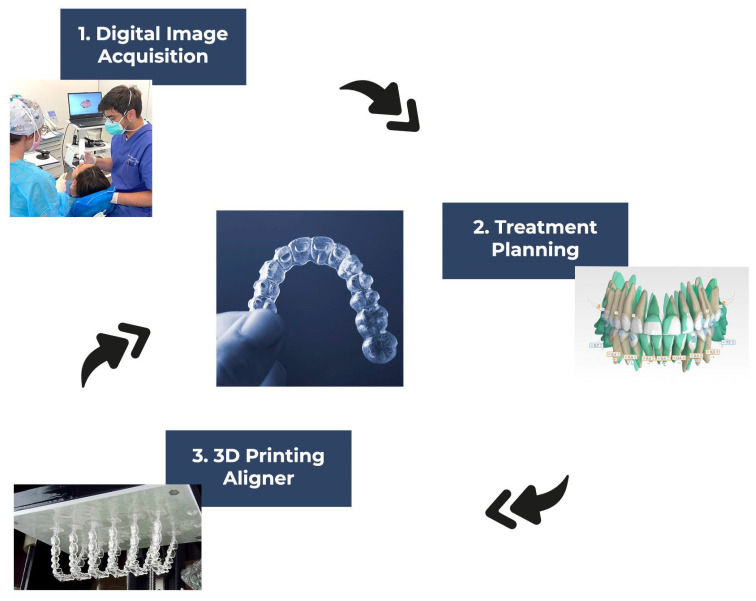
The stepwise fabrication process of 3D direct printed clear aligners.

In dental and medical modelling, 3D printing, also known as additive manufacturing, is now reaching a level of maturity (Figure 2). It dates back to the 1980s.

There are multiple applications of 3D printing in oral surgery, prosthodontics, restorative dentistry, orthodontics, implantology, and instrument manufacturing. Three-dimensional printing allows the manufacturing of pieces layer by layer, unlike standard manufacturing methods that rely on machining, molding, and subtractive methods.

The materials currently used for 3D printing in orthodontics include acrylonitrile butadiene styrene plastic, stereolithography material (epoxy resins), polylactic acid, polyamide (nylon), glass-filled polyamide, silver, steel, titanium, photopolymers, wax and polycarbonate (PC) [20].

## 6. Reduce, Reuse, Recycle, Rethink

In 2017, the World Dental Federation published the document ‘Sustainability in Dentistry’ based on the United Nations’ 2030 Agenda for Sustainable Development.

Two authors, among the selected articles, discuss the possibility of applying these principles in the field of dentistry as well. Dental clinics should also promote a transition towards a sustainable economy [21,22].

As proposed by the Department of Economic and Social Affairs of the United Nations, the concept of the 4Rs, reduce, reuse, recycle, and rethink, is a strategy that should also be adopted by healthcare professionals, particularly dentists. Even the behaviors and advice given to patients can help achieve these goals, encouraging patients to use eco-friendly products for oral hygiene at home, for example [23].

In dentistry, it is essential to minimize resource consumption to reduce waste generation. We need to re-evaluate the operational strategies employed in dental practices, with a greater focus on sustainability.

**Figure 3:**
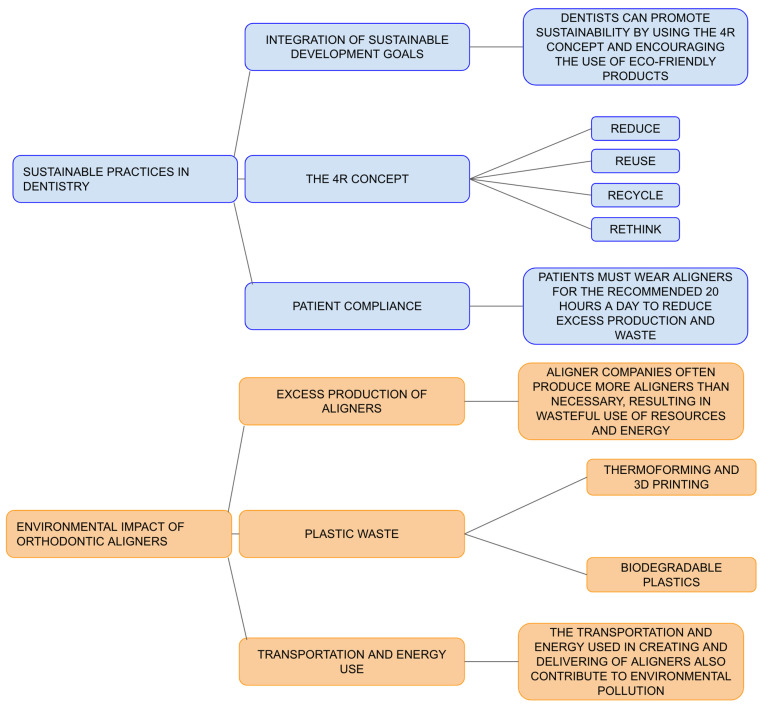

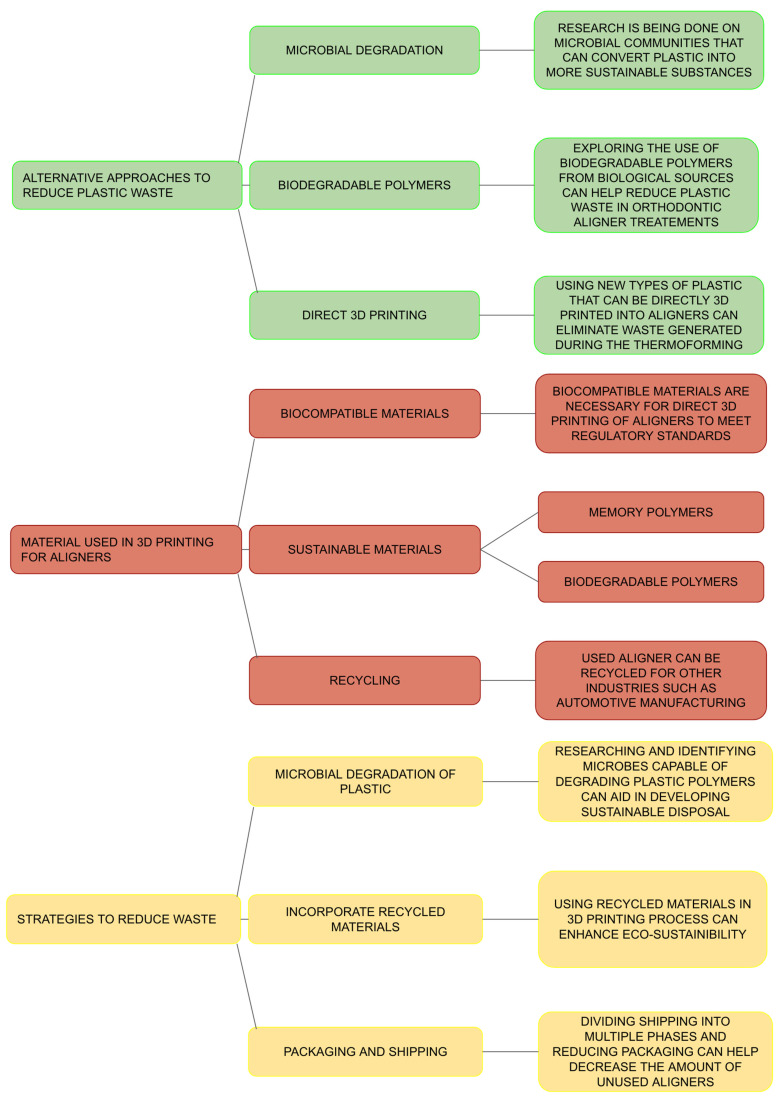

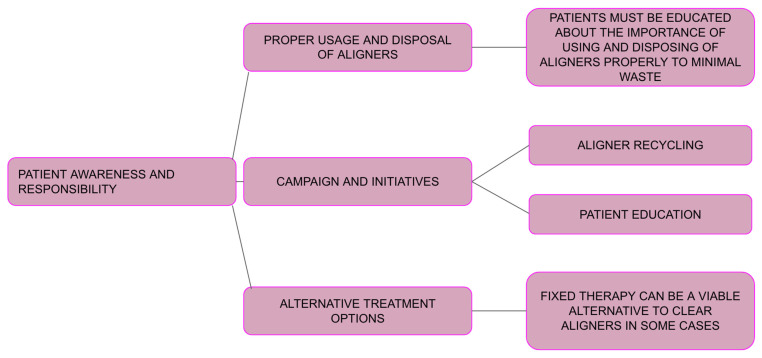
Conceptual map for sustainable orthodontics.

In particular, the 4Rs concept could be applied in the field of orthodontics. Figure 3 illustrates a conceptual map outlining the strategy that could be used in all clinical phases.

In particular, in the construction of orthodontic aligners, the use of these concepts is of fundamental importance.

Considering that each aligner can move a tooth up to 0.25 mm, more aligners are needed for complex cases. After the orthodontist creates and approves a virtual treatment plan, the aligner distributor manufactures the complete set of aligners at once. Simple cases usually require 7 to 20 aligners per arch, while complex cases may need 50 or more. Patients typically switch aligners every 1–2 weeks, so a full set of 70 aligners would last at least 18 months.

If adjustments to the treatment plan are necessary, additional aligners can be ordered for refinement. Should the orthodontist need to alter the strategy, treatment can be paused, new aligners ordered, and any unused aligners discarded. This flexible approach accommodates various situations, such as lost aligners, non-compliant patients, or unexpected dental procedures. It benefits both the orthodontist and the patient, as the distributor ensures the correction of malocclusions regardless of the number of aligners required.

It is important to recognize that fixed therapy can be an effective alternative, especially when aligners might extend treatment times excessively. Dental movements with aligners may not always be as predictable as expected, and patients may not consistently wear their aligners for the recommended 20 h per day. This leads to the production of more aligners than are actually used, resulting in wasted resources and energy. Although distributors are aware of this overproduction, reducing it does not seem to be a priority.

It is common to discard a significant number of unused aligners, along with their plastic cases, covers, and various packaging materials. Companies could mitigate this waste by not sending all of a patient’s aligners at once and instead allowing the clinician to specify the quantity of aligners needed at a given time [24,25].

Tartaglia et al. have emphasized the lack of reliable data regarding the environmental impact of waste generated while producing invisible aligners beyond the aligners themselves. This includes plastic waste used for 3D models and waste discarded after thermoforming the aligners. The environmental impact of these additional materials exceeds that of the aligners alone. Three-dimensional printing is the leading technology utilized for manufacturing orthodontic models.

This technique involves thermoforming a series of clear aligners into physical three-dimensional prints, which are finished and polished. The process is time consuming, labor intensive, and costly, depending on factors such as the type of malocclusion, the aligner brand, and associated protocols.

The authors propose that incorporating recycled materials for 3D printers could enhance the eco-sustainability of 3D printing [20,21]. Recycled and recyclable materials could be used and incorporated in the printed models.

As in other commercial fields, the location of production for orthodontic aligners is of fundamental importance. It is necessary to consider the pollution caused by transportation, as well as the energy used in their creation and, especially, in their disposal.

In two articles, Paszkiewicz, S et al. and Temoor Ahmed et al. [26,27] discuss the possibility of producing materials that do not need to be disposed of because they are biodegradable.

Although biomaterial technologies are continuously evolving, further studies are essential to evaluate the elasticity coefficients of these materials. However, biodegradable plastics in addressing malocclusions will be considered as one approach to mitigate the environmental impact of traditional plastic materials in the future [26,27].

Berlinda et al. focus on identifying and characterizing microbes capable of degrading different plastic polymers. Microbial communities can convert specific plastics into more manageable substances through aerobic and anaerobic mechanisms. Therefore, exploring the synthesis of biodegradable polymers derived from biological sources is crucial in investigating novel microbial strains and degradation mechanisms for fossil-based polymers [28].

Another approach to reducing plastic waste in orthodontic aligner treatments is using new types of plastic that can be directly 3D printed into clear aligners, thus eliminating waste generated during the thermoforming and 3D printing of physical models [29].

At this moment, the amount of plastic generated by physical models is totally unknown, despite the types of materials used and their frequent use.

However, for the direct 3D printing process, only a few commercially available materials meet biocompatibility standards, aesthetics, and mechanical properties. The first material introduced for 3D direct printing of aligners is Tera Hard TC-85, Graphy, Seoul (Republic of Korea), which has obtained approvals from regulatory bodies such as the Korea Food and Drug Administration (KFDA), European Commission (EC), and Food and Drug Administration (FDA), as stated on the company’s website [30].

Another noteworthy brand is 3Dresyns, Barcelona (Spain), which offers resins containing 30–40% renewable bio-based ingredients. It is the first and only global 3D resin supplier to sell “monomer-free” biocompatible 3D resins, colors, and additives with Material Safety Data Sheets (MSDSs) indicating non-toxic raw materials and without any toxicity risk hazard pictograms [31].

Some companies in the United States and Europe have initiated patient awareness campaigns regarding the correct disposal and recycling methods for aligners. For instance, ‘Impress’ has launched an aligner recycling initiative, placing collection containers in all their flagship locations. Patients can visit these locations to dispose of their used aligners, which the company will collect for recycling or use as fuel for heat or electricity generation.

These aligners can be recycled, unfortunately, not to build new aligners and create a circular economy, but to be repurposed in other industries, such as in automotive manufacturing or to create soundproofing slabs or floors.

Patients can participate in aligner recycling by depositing their waste at affiliated dental practices, which will then send the aligners to TerraCycle, Trenton, NJ, (USA), a global waste recycling organization [11,12].

Nightingale C. describes some consideration outlined at the second UKI Invisalign Summiti. She pointed out how, unfortunately, some initiatives are limited to designated drop-off locations or raising awareness. Still, they fail to address the significant number of patients who struggle with the surge in aligner usage and the resulting global plastic pollution. Therefore, the responsibility to find comprehensive solutions remains imperative for the widespread use of plastic materials [32].

Elshazly et al. suggest that memory polymers could contribute to using fewer aligners. A single aligner produced with these materials could replace three aligners manufactured with conventional materials by allowing shape change within the same aligner. Reducing the number of aligners used for each treatment would decrease plastic waste and the energy consumption associated with their production [33].

Despite the various options available to reduce the environmental impact of orthodontic treatments with transparent aligners, the orthodontic market appears reluctant to explore alternatives to traditional therapy with thermoformed aligners. Instead, they promote clear aligners, allowing dentists to keep ordering them until they achieve the desired result. At this point, both the dentist and the patient play an increasingly crucial role in minimizing the environmental impact of these treatments [34].

Of course, it is also the orthodontist’s responsibility to make proper use of this system and to educate their patients on the importance of using the aligners properly and not throwing away the used aligners.

## 7. Conclusions

In conclusion, it is evident that there is a pressing need for increased awareness surrounding the issues discussed.

More studies are needed to understand the consequences of NMPs on the human body. More studies are needed to quantify the amount of NMPs that may be produced from the fabrication and application of these aligners, and recognizing the environmental impact of failed prints and post-processing waste is crucial.

Moreover, understanding the benefits of fixed therapy as a viable alternative treatment option, particularly in cases where aligners may prolong treatment unnecessarily, is important.

Educating patients on the proper usage and disposal of aligners is essential to minimize waste.

Implementing strategies such as dividing shipments into multiple phases and reducing packaging can help decrease the number of unused aligners.

Additionally, incorporating recycled materials into 3D printing processes can enhance eco-sustainability.

Exploring new types of plastic that are suitable for 3D printing clear aligners, such as biodegradable or environmentally friendly polymers, is advisable. Furthermore, researching and identifying microbes capable of degrading plastic polymers can aid in developing sustainable disposal methods.

Campaigns promoting correct disposal and recycling techniques for aligners should be launched, with collection containers placed in flagship locations for convenient recycling. Companies can collect used aligners for recycling or utilize them as a source of fuel for heat or electricity generation. By recycling and repurposing aligners, we can contribute to a more sustainable future and reduce the environmental impact of orthodontic treatment.

## Data Availability

The original contributions presented in the study are included in the article, further inquiries can be directed to the corresponding author.
